# Study protocol and rationale of “the UP project”: evaluating the effectiveness of active breaks on health indicators in desk-based workers

**DOI:** 10.3389/fpubh.2024.1363015

**Published:** 2024-03-19

**Authors:** Carlos Cristi-Montero, Ricardo Martínez-Flores, Juan Pablo Espinoza-Puelles, Laura Favero-Ramirez, Natalia Zurita-Corvalan, Ignacio Castillo Cañete, Jaime Leppe, Gerson Ferrari, Kabir P. Sadarangani, Jorge Cancino-López, Sam Hernandez-Jaña, Tuillang Yuing Farias, Vanilson Batista Lemes, Fernando Rodríguez-Rodríguez, Caroline Brand

**Affiliations:** ^1^IRyS Group, Physical Education School, Pontificia Universidad Católica de Valparaíso, Valparaíso, Chile; ^2^School of Physical Therapy Faculty of Medicine, Clínica Alemana, Universidad del Desarrollo, Santiago, Chile; ^3^Universidad de Santiago de Chile (USACH), Escuela de Ciencias de la Actividad Física, el Deporte y la Salud, Santiago, Chile; ^4^Facultad de Ciencias de la Salud, Universidad Autónoma de Chile, Providencia, Santiago, Chile; ^5^Universidad Autónoma de Chile, Santiago, Chile; ^6^Escuela de Kinesiología, Facultad de Salud Y Odontología, Universidad Diego Portales, Santiago, Chile; ^7^Laboratorio de Fisiología del Ejercicio y Metabolismo, Escuela de Kinesiología, Facultad de Medicina, Universidad Finis Terrae. Santiago, Santiago, Chile; ^8^Escuela de Kinesiología, Universidad Santo Tomas, Viña del Mar, Chile; ^9^Universidade Federal do Rio Grande do Sul, Escola de Educação Física, Fisioterapia e Dança, Porto Alegre, Brazil

**Keywords:** sedentary behavior, physical activity, interventions, occupational health, cardiometabolic risk

## Abstract

**Background:**

Excessive sedentary time has been negatively associated with several health outcomes, and physical activity alone does not seem to fully counteract these consequences. This panorama emphasizes the essential of sedentary time interruption programs. “The Up Project” seeks to assess the effectiveness of two interventions, one incorporating active breaks led by a professional and the other utilizing a computer application (self-led), of both equivalent duration and intensity. These interventions will be compared with a control group to evaluate their impact on physical activity levels, sedentary time, stress perception, occupational pain, and cardiometabolic risk factors among office workers.

**Methods:**

This quasi-experimental study includes 60 desk-based workers from universities and educational institutes in Valparaiso, Chile, assigned to three groups: (a) booster breaks led by professionals, (b) computer prompts that are unled, and (c) a control group. The intervention protocol for both experimental groups will last 12 weeks (only weekdays). The following measurements will be performed at baseline and post-intervention: cardiometabolic risk based on body composition (fat mass, fat-free mass, and bone mass evaluated by DXA), waist circumference, blood pressure, resting heart rate, and handgrip strength. Physical activity and sedentary time will be self-reported and device-based assessed using accelerometry. Questionnaires will be used to determine the perception of stress and occupational pain.

**Discussion:**

Governments worldwide are addressing health issues associated with sedentary behavior, particularly concerning individuals highly exposed to it, such as desk-based workers. Despite implementing certain strategies, there remains a noticeable gap in comprehensive research comparing diverse protocols. For instance, studies that contrast the outcomes of interventions led by professionals with those prompted by computers are scarce. This ongoing project is expected to contribute to evidence-based interventions targeting reduced perceived stress levels and enhancing desk-based employees’ mental and physical well-being. The implications of these findings could have the capacity to lay the groundwork for future public health initiatives and government-funded programs.

## Background

Sedentary behavior is defined as any waking behavior with an energy expenditure of ≤1.5 METs while sitting, reclining, or lying down (1). This behavior has been negatively associated with various health markers, such as, sarcopenia, osteopenia, diabetes, hypertension, metabolic syndrome, cardiovascular disease, overweight, obesity, and stress (2–7). This scenario is even worse in desk-based workers exposed daily to a high amount of sedentary time, decreased work performance, and increased absenteeism which implies unfavorable implications for the worker, company, and society (8). Consequently, interrupting sedentary time with physical activity emerges as a valuable strategy for addressing sedentary behavior (9–11).

In this sense, advocating for workplace programs that promote physical activity and reduce the prolonged time spent in sedentary activities becomes a practical proposition in occupational health (involving both physical and mental aspects of well-being). The World Health Organization characterizes occupational health as a highly interdisciplinary effort to safeguard and enhance workers’ health by preventing and managing work-related diseases and accidents (12). A key point to emphasize is that scientific evidence underscores how certain detrimental health outcomes associated with sedentary behaviors seem to be independent of individuals’ physical activity levels (13). Thus, interventions based on physical activity alone would not be entirely effective in counteracting the adverse effects of sedentary behavior on health (9). In this regard, strategies for reducing sedentary time have been associated with decreased body fat, increased fat-free mass, decreased abdominal fat, resting heart rate, and blood pressure (10, 14–16).

Given the potential hazards associated with prolonged sitting at work and physical inactivity, including increased stress and occupational pain (17–19), it is imperative to examine workplace interventions that can mitigate these behaviors and promote overall health and well-being (20). The literature has mainly proposed two types of active breaks to interrupt sedentary behavior in desk-based workers: “Booster Breaks” (B-B) and “Computer Prompts” (C-P) (21–23). On the one hand, B-B can be defined as guided, programmed, and performed active breaks in the workplace, with an average duration around of 15 min (21). This type of break benefits individuals’ physical and mental health by reducing stress, improving social interactions at work, increasing physical activity levels, and decreasing body mass index (BMI) (21, 22). On the other hand, C-P is characterized by unguided breaks performed through the software on the work computer, with a short duration (e.g., 2–3 min), accumulating around 15 min during the whole working day (21). Two systematic reviews with meta-analyses have concluded that this type of break can reduce sedentary behavior and increase physical activity levels, but the body of evidence comparing B-B and C-P is still limited (23, 24).

Most of the research on workplace interventions to reduce sedentary behavior has been conducted in high-income developed countries (21, 23, 25). In Latin America there is very little information on this topic, specifically in Chile, to our knowledge there are no previous studies on strategies to reduce sedentary time at work. However, previous research indicates that in 2016, 35.9% of the Chilean adult population spent more than 4 h in sedentary time (26). Thereby, the current project intends to fill gaps in the existing literature on this topic in an under-researched geographical world region. First, previous studies investigating B-B and C-P protocol parameters used physical activity questionnaires, pedometers, BMI, and waist circumference measures to analyze physical activity levels and body composition, respectively, (21, 23). However, to date, scarce evidence has evaluated movement behavior by accelerometry and dual-energy X-ray absorptiometry (DXA) to accurately measure participants’ body composition using the same protocol. DXA provides comprehensive body composition assessment, including fat mass, lean mass, and bone mineral density, for a more accurate portrayal of participants’ health (27, 28). Accelerometry delivers precise, device-based measurements, revealing daily movement patterns with minimal reliance on self-reporting (29).

Second, this study introduces an innovative approach by contrasting two types of interventions (B-B and C-P), which will be equivalent in duration and intensity. However, these interventions differ in their structures. B-B will be conducted continuously for 14–16 min, while C-P will consist of brief 2-min breaks scattered throughout the 8-h workday (averaging 14–16 min). By assessing the differentiated effects of these breaks, we aimed to identify effective strategies to improve the health of office workers.

Third, previous scientific evidence has shown that diverse variables can affect our main outcomes, encompassing factors such as sleep time and quality, socioeconomic status, smoking, and eating habits (30–32). Consequently, it is imperative to explore these interrelated factors using a more comprehensive approach (33). Initially, these variables will be used as covariates to explore their influence on study outcomes.

Finally, it is crucial to take public health measures to contribute to the reduction of sedentary behaviors in workers. Thus, we anticipate that our findings could contribute to the formulation of evidence-based interventions that can be deployed or not to improve the health and well-being of this population.

Therefore, this manuscript aims to describe the design and methods of “The Up Project,” which seeks to establish the efficacy and differences between two interventions addressed for interrupting sedentary time in desk-based workers. The principal outcomes will include variations in cardiometabolic risk factors, sedentary and physical activity levels, perception of stress, and occupational pain. Accordingly, our primary hypothesis is that both intervention groups will yield better results compared to the control group. However, we expect no discernible differences between the intervention groups due to their equivalence in duration and intensity. This is mainly because previous studies suggest that varying the frequency of breaks can improve cardiometabolic risk markers. However, studies comparing different frequencies at the same intensity and volume are lacking (34–36). In this sense, previous literature suggests that when exercise or physical activity volume and intensity are kept constant, frequency does not significantly impact muscle mass or cardiovascular risk (37, 38).

## Methods

### Study design and ethical considerations

“The Up Project” comprises a quasi-experimental study in which the sample will be selected through cluster sampling, with workplaces serving as the basis for establishing both interventions and control groups (ClinicalTrials.gov identifier: NCT05844267). This project has been approved by the Ethics Committee of Pontificia Universidad Católica de Valparaíso (BIOEPUCV-HB 580–2023) and will be conducted following the Declaration of Helsinki and the guidelines of the SPIRITS (Standard Protocol Items: Recommendations for Intervention Trials) checklist (39). Additional information is provided in the Supplementary material. Written consent will be obtained from the participants before beginning and will be guarded and stored by the principal investigators (C.C-M. and CB) to maintain the confidentiality of the participants. All protocol modifications will be communicated and registered on ClinicalTrials.gov.

### Training of personnel and quality control

Our research group will organize training sessions for investigators and key personnel prior to data collection. Quality control and rigorous standardization of measurement protocols across centers are critical for the success of intervention studies (40). All investigators from our research group shared responsibility for quality control. The principal investigators will oversee regulatory compliance, protocol adherence, data accuracy, personnel training, and regulatory document management. During data entry, key variables will be checked for accuracy with assigned interval controls. A review will be required for any data entered outside the preset ranges. The principal investigators (CC-M and CB) make the final decision to terminate the trial.

### Sample recruitment

Desk-based workers (60 in total) will be recruited voluntarily from universities and educational institutes in the Valparaiso region, Chile. We will extend an open invitation to all desk-based workers of the Pontificia Universidad Católica de Valparaíso (PUCV) in the Faculty of Philosophy and Education and the central office located in the Central Campus. The control group sample will be obtained from Naval Academy’s desk-based workers. It is important to note that the participants of the control group are civilians or retired marines who do not undergo any type of military training or physical tests. Subsequently, our research team will be visiting their offices to conduct face-to-face recruitment. During this process, the participants will be provided a detailed description of the scientific background, research objectives, and safety measures by our research group. We aim to ensure that all participants clearly understand our research and feel comfortable participating in our study. Once accepted, they will be assigned to a group according to the work site. Upon completion of the study, the participants will be given a full report on changes in body composition, physical activity levels, and sedentary time. After the data collection period, the control group will have the opportunity to participate in the same intervention program.

#### Inclusion and exclusion criteria

In this study, participants will need to be desk-based women and men workers in order to be included, between 18 and 60 years of age, who belong to an educational institution in the Valparaiso region, with a full-time contract.

As exclusion criteria will be considered participants who do not have a full-time job, individuals with physical limitations for physical exercise or undergoing weight loss treatment, pregnant and lactating (in the first 6 months postpartum) women (41), or pacemaker users (42). In addition, participants with a participation frequency lower than 70% in the protocol will not be considered; however, a sensitivity analysis will be performed based on the intention-to-treat principle (43).

#### Sample size calculation

The sample size was calculated using the G*Power software. This calculation considered the repeated measures of factorial analysis of variance (ANOVA), with an effect size (f) of 0.20, alpha value of 0.05, and beta of 0.80. The study involved three groups and two evaluation time points (pre/post), with an estimated follow-up loss of 10%. Consequently, 60 participants will be distributed as 20 participants per group: two experimental groups (B-B led by professionals and unled C-P) and one control group. The groups will be sex balanced because of the predominance of women in this type of work. In this sense, for selecting participants, we used the sex ratio of workers in group B-B (the lowest men proportion). In this site, there is a proportion of 27.1% of men and 72.9% of women; thus, our sample will try to approach this proportion in the two remaining groups.

#### Procedures

**Figure 1:**
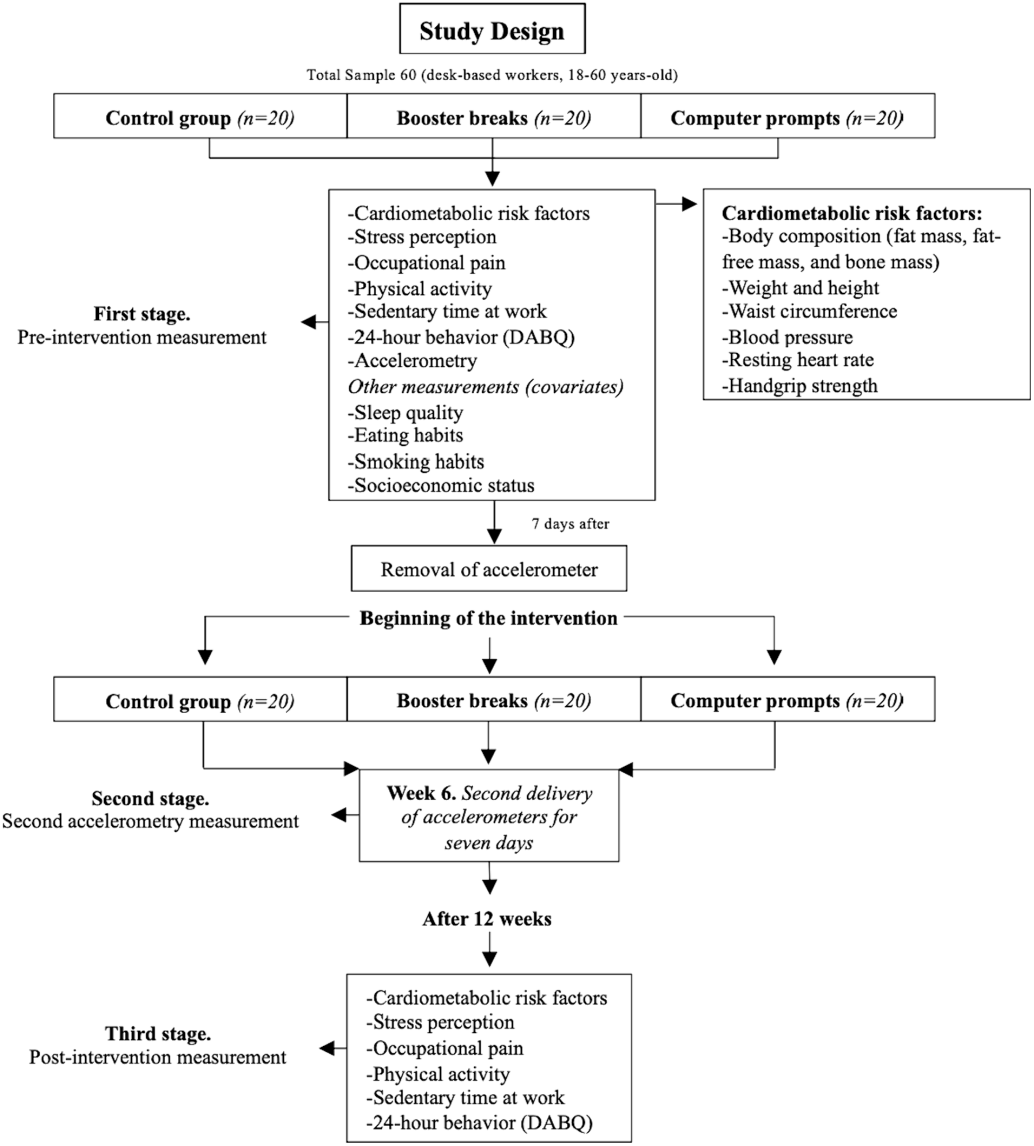
Study design. DABQ, Daily Activities Behavior Questionnaire.

The project will be conducted in three stages, comprising two visits to the Physical Performance and Health Laboratory of the PUCV (stage one and stage three) and one visit to the participants’ offices (stage two). Previous to measurement sessions, participants will be asked not to consume any type of stimulant prior to the measurements (such as caffeine or tea, mainly for blood pressure and heart rate). The first stage will consist of a pre-intervention measurement session in our laboratory, in which cardiometabolic risk factors, stress perception, occupational pain, physical activity, sedentary time at work, 24-h behavior, eating and smoking habits, and sociodemographic information will be evaluated. In addition, participants will be given an accelerometer, which will be removed 7 days later, and the intervention will begin once the accelerometer is removed. The intervention will have a total duration of 12 weeks, because previous literature indicates that 12 weeks is sufficient time to observe behavioral changes that can be extended beyond the time of the intervention (44). In the second stage (sixth week of intervention), the three groups will receive accelerometers to re-evaluate their daily physical activity intensity, sedentary time, and breaks in sedentary time. Finally, the third stage (post-intervention measurement) will consist of a second visit to our laboratory, where all variables will be re-evaluated. The anonymized and raw data obtained will be made available by the corresponding author upon reasonable request. A diagram of the study design, sample, and measures is presented in Figure 1.

### Main variables

In the first stage, upon arrival at the laboratory, participants will be asked to read and sign an informed consent form and then begin the measurements. First, the participant will be asked to complete questionnaires (stress perception, occupational pain, physical activity, sedentary time at work, 24-h behavior, eating habits, smoking habits, and socioeconomic status). Subsequently, the participants will be asked to enter the DXA room in the laboratory, where their height, weight, and waist circumference will be assessed. Once these measurements have been taken, the participant will be asked to lie on the scanner table for analysis. Upon completion of the scanner (estimated duration of 7 min), the participant’s resting heart rate will be determined while they remain in a lying down position. Thereafter, the individual will be requested to sit down, and their blood pressure will be measured in their left arm twice consecutively. After this, handgrip strength will be measured 2 times for each hand. Lastly, the accelerometer will be provided to the participant for a period of 7 days, for the initial measurement.

In the second stage, our research team members will go to the participants’ workplaces for the second delivery of accelerometers for 7 days. In the third stage, participants will be administered questionnaires (stress perception, occupational pain, physical activity, and sedentary time at work). Subsequently, measurements of cardiometabolic risk factors will be made with the previously mentioned protocol. All measurements will be carried out between 16:00 and 20:00 for the convenience of the participants due to their work schedule. Is important to note that all the factors chosen in this work are relevant for public health research since they are social determinants of importance for the design of public policies in our specific population.

#### Cardiometabolic risk factors

DXA scans (General Electrics, model Lunar, series 212,069, California, United States) will be used to assess body composition (fat mass, fat-free mass, and bone mass). We will use a digital balance (OMRON HN-289-LA, Kyoto, Japan) and a portable stadiometer (SECA, model 213, GmbH, Hamburg, Germany) to measure weight and height. After exhaling, waist circumference will be measured using a Lufkin metallic tape measure (W606PM, Ohio, United States) three times by the same evaluator, and the smaller measurement will be valid (45). Additionally, we will use a digital monitor (model HEM-7120, OMRON Kyoto, Japan) to measure blood pressure twice (an average will be used for the analysis), and a pulse oximeter (model Prince-100B5, Heal Force, Shanghai, China) to determine the resting heart rate. Finally, we will measure handgrip strength using a digital dynamometer (JAMAR Plus Digital Hand Dynamometer, Illinois, United States). This will be evaluated two times per hand, with the person standing and the arm extended parallel to the body. The maximum measurement per hand and an average of both measurements may be used for analysis (46). Previous studies indicate that these measurements are related to the relative risk of cardiometabolic factors (30–32, 47).

#### Physical activity, sedentary time, and sleep time

An ActiGraph GT3X + accelerometer (ActiGraph, Illinois, United States) will be utilized for device-based measurement to monitor physical activity, sedentary behavior, and sleep time. The accelerometer has been validated for assessing physical activity, sedentary behavior, and sleep quality (48, 49).

Participants will be instructed to continuously wear the device on their non-dominant wrist for 7 days (24 h/day) and only remove it for water activities. Wrist-worn accelerometers have become popular because they place a low burden on participants and provide valid and reliable measurements of physical activity (50). Accelerometers will be initialized to a sampling rate of 100 Hz and downloaded using ActiLife^®^ software (ActiGraph, Pensacola, FL). The raw accelerometer data (.gt3x) will be processed using the GGIR 3.0–5 package (51), in R studio. The minimum amount of accelerometer data that will be considered acceptable will be 600 min of valid daily monitor wear on at least 4 days including a weekend day (52, 53). The MVPA threshold and cutoff points used will be those proposed by Hildebrand et al. (54, 55). Sleep time (periods without z-angle changes of >5° for at least 5 min) will be calculated using an algorithm proposed by van Hees et al. (56), and identification of onset and wake time will be guided by the HDCZA algorithm (51). All participants start with their pre-intervention and post-intervention measurements on the same dates, in order to maintain the same measurement conditions for all and reduce the risk of bias.

To complement the accelerometer data, we will administer four questionnaires: (a) the Physical Activity Questionnaire (GPAQ), a standardized tool recognized by the World Health Organization that provides valuable information on physical activity levels globally (57), (b) a single physical activity question to identify the level of physical activity through a single item (58), (c) the Occupational Sitting and Physical Activity Questionnaire (OSPAQ) will be used to determine the proportion of time participants spend on sedentary behavior during work hours (59), and finally, (d) the Daily Activities Behavior Questionnaire (DABQ) to gather additional information on participants’ physical activity, sedentary behavior, and sleep habits over 24-h (60). Questionnaire will also be used to categorize participants as non-participants in physical exercise programs according to our exclusion criteria. Questionnaires b, c, and d will be validated in Spanish in a parallel study by our investigation group. The combination of device-based measurement tools and standardized questionnaires will enable an accurate and comprehensive assessment of physical activity levels, sedentary behavior, and sleep quality. All of these variables will allow us to analyze the effect of reducing sedentary time and its impact on the study’s objective (61, 62). The data collected will be crucial for analyzing the results and effectiveness of interventions among office workers.

#### Stress perception

The Perceived Stress Scale Questionnaire (PSS-14) will measure stress perception in various situations. This questionnaire was initially validated by Cohen, Kamarck, and Mermelstein in 1983 (63) and has been adapted for use in Chile by Erik Marín (64). The PSS-14 questionnaire assesses an individual’s perception of different situations in which their stress levels may be influenced. The questions focused on the last month and are rated on a Likert scale ranging from 0 to 4 (0 = never, 1 = rarely, 2 = sometimes, 3 = often, 4 = very often). To obtain the total PSS score, the scores of items 4, 5, 6, 7, 9, 10, and 13 are reversed (0 = 4, 1 = 3, 2 = 2, 3 = 1, 4 = 0), and then the 14 items are summed. A higher score indicates a higher level of perceived stress.

#### Occupational pain

The Nordic Musculoskeletal Questionnaire, a standardized tool recommended by the Institute of Public Health of the Chilean Ministry of Health, will measure occupational pain. This questionnaire is specifically designed to detect musculoskeletal symptoms related to desk-based work, including pain in the neck, shoulder, thoracic spine, wrist, lumbar spine, hip, knee, and ankle (65). This questionnaire uses closed-ended (yes or no) questions regarding the prevalence of pain in the aforementioned joints, which facilitates obtaining dichotomous pain categories per joint, per participant. With the implementation of this tool, we will be able to assess both the general and specific pain experienced by participants in the workplace. This method provides a comprehensive evaluation of the prevalence and severity of musculoskeletal pain, enabling a thorough analysis of the overall impact on employees’ well-being (18, 66, 67).

#### Other measurements (covariates)

Previous studies have established that cardiometabolic risk factors are not only affected by body composition, physical activity, or sedentary behavior but can also be influenced by various healthy lifestyle habits (30–32). Therefore, we identified a series of related factors that will be included as covariates in our statistical analyses to assess their impact on our outcomes.

We will use an ActiGraph accelerometer to measure sleep quality (48, 49). This variable plays a fundamental role in cardiovascular health (68). Lack of quality sleep can increase blood pressure and inflammation by increasing cortisol levels and decreasing immunity, elevating the risk factors for cardiovascular problems (68).

The questionnaire on adherence to the Mediterranean diet will be applied to assess eating habits, specifically designed to measure participants’ adherence to it and determine its relationship with cardiometabolic factors (69–71). This questionnaire gathers information on the frequency of consumption of foods characteristic of the Mediterranean diet using a Likert scale format. To assess participant adherence, the score of each question is summed to obtain a total score, reflecting the individual’s adherence to the Mediterranean diet.

The Fargerstörm test, which scores various aspects of smoking habits on a Likert scale and enables us to determine the level of dependence on this substance, will be used to assess smoking habits (72).

Furthermore, we will obtain information on the participants’ socioeconomic status using a questionnaire on the distribution and average autonomous income of households by income deciles. This questionnaire provides essential data on the *per capita* income of the participants and follows the guidelines of the “Asesoría Técnica Parlamentaria” of the Chilean government (73). In addition, the educational level and occupational status of the participants will also be recorded. These measures are considered relevant as predictive cardiometabolic risk factors (70, 74, 75). In addition, sociodemographic questions will be asked to characterize the study sample.

### Protocol interventions

#### Computer prompts group (unled breaks)

Active breaks during the workday will be guided by an application called “Ponte de Pie por tu Salud” developed by the Chilean Ministry of Health.^1^ The exercises included in these breaks focus on stretching, mobility, and muscular resistance with light to moderate intensity (i.e., Rate of Perceived Exertion [RPE] 3–4) following the Chilean Safety Association (*Asociación Chilena de Seguridad*—ACHS) recommendations (76, 77). An example of the exercises to be performed can be found in the following link (See Footnote 1). The sessions will begin with a warm-up and end with a cool-down, following the ACHS guidelines. The application is configured to generate 2-min breaks every hour for 8 h per day, ensuring that participants accumulate an average of 14–16 min of active breaks each day.

#### Booster breaks group (led breaks)

We will implement active breaks lasting approximately 14 to 16 min every working day for 12 consecutive weeks, for five workdays, excluding weekends. By implementing this approach, we can ensure that both interventions are distributed equally over time. These breaks will consist of breathing exercises, stretching exercises, and exercises recommended by the ACHS (76, 77). They will be similar in intensity to exercises proposed in the “Ponte de Pie por tu Salud” application. Each session will be divided into three parts: warm-up (2 min), main work (12 min), and cool down (1 min). The intensity of the exercises will range from light to moderate (RPE 3–4), and at the end of each session, we will evaluate intensity perception using Borg’s scale (78). Trained personnel from our research team will conduct all the sessions throughout the intervention. The intervention will be conducted in small groups (4–5 participants). While this may have a different impact on stress or motivation than the computer prompts group breaks, this is a limitation inherent to the type of intervention (22).

During the warm-up period, we will include breathing and muscle activation exercises that will last approximately 2 min. For the main work, we will target four main muscle groups: neck and shoulders, arms and wrists, lumbar back and hips, and knees and ankles, which will be distributed and combined according to Table 1.

**Table 1:** Graphical example of the time distribution of muscle groups in 1 month of intervention.

Days	Week 1	Week 2	Week 3	Week 4
Monday	i 70% - ii 30%	ii 70% - i 30%	i 70% - ii 30%	ii 70% - i 30%
Tuesday	iii 70% - iv 30%	iv 70% - iii 30%	iii 70% - iv 30%	iv 70% - iii 30%
Wednesday	i 70% - iii 30%	iii 70% - i 30%	i 70% - iii 30%	iii 70% - i 30%
Thursday	ii 30% - iv 70%	iv 30% - ii 70%	ii 30% - iv 70%	iv 30% - ii 70%
Friday	iv 30% - i 70%	i 30% - iv 70%	iv 30% - i 70%	i 30% - iv 70%

B-B, Booster Breaks group; (i) neck and shoulders, (ii) arms and wrists, (iii) lumbar back and hips, and (iv) knees and ankles. The program will be cycled over the next 2 months. Please refer to the Supplementary material for more information on the session and exercises to be implemented.

To prevent participant dropout and maintain motivation throughout the intervention, both variations and combinations of the main muscle groups to be worked on will be introduced. The two muscle groups will be combined daily, with 70% of the time allocated to the first group and 30% to the second group. The order and time percentages will be reversed the following week. In the second month, elastic bands and canes will be incorporated to support the exercise performance. The main work session will last nearly 12 min.

It is important to note that although B-B includes exercises that differ from those of the application, the intensity will be the same. Borg’s scale will be employed for this purpose to ensure comparability between the interventions. In this sense, our investigation group conducted a pilot test of the selected exercises to determine the correct intensity in relation to the exercises proposed by the application. In addition, the staff in charge of conducting the group B-B active breaks ensured that the sessions were performed between light and moderate intensity (i.e., RPE 3–4). Participants played an indirect role in the validation of the intervention’s B-B component. Although no focus group was conducted to directly gather their input for the intervention’s design, which is based on government programs, their well-being at work was taken into account when creating the exercise plans (for instance, the subjects did not wish to perspire, so the exercises were of mild to moderate intensity). Furthermore, it should be emphasized that the participants in both intervention groups are affiliated with the institution where the principal investigators are employed, thus providing a known context for the participants.

#### Control group

The control group will not have any type of intervention; they will be asked to continue with their normal life habits. At the end of the study, participants will be invited to participate in a physical activity program at their workplace.

#### Follow up of participants

For the C-P group, the computer application will record the number of times the breaks will be accepted, rejected, or postponed, and it will generate a weekly summary. Furthermore, a member of our research group will consistently make phone calls to ensure that everything remains in order. In the case of the B-B group, all sessions will be recorded, with attendance tracking for all participants. Meanwhile, for the control group, a research group member will periodically remind them of the recommendations provided at the beginning of the 12 weeks. After the intervention, participants will be compensated by receiving a report with the values provided by accelerometry and a complete report of their body composition from the DXA measurements. There will be no monetary compensation. In addition, to qualitatively analyze the participants’ experience with the interventions, general and specific questions will be asked about the performance of the breaks (C-P and B-B). These questions will be asked in a private questionnaire format so as not to influence the answers of the participants. These results will be analyzed as a secondary objective in order to identify barriers and facilitators to be considered in future studies. For more information on these questions consult Supplementary material.

In case of desertion for any reason (i.e., leaving the country, change of job, impossibility of measurements), a sensitivity analysis will be performed with and without intention to treat. It is important to note that the exercise protocol was based on previous recommendations by Chilean governmental organizations, and that the protocol is designed to pose little or no risk to the health of the participants. However, in case of any type of injury risk, the participants’ institutions have medical insurance that will be effective if necessary.

### Data analysis plan

Owing to the characteristics of the study (i.e., cluster for conformation of the groups), allocation concealment was not possible. However, the blinding of the evaluator is detailed in the study records (more information in ClinicalTrials.gov, identifier: NCT05844267).

Descriptive data from participants will be summarized and presented as means and standard deviations for continuous variables, and frequencies and percentages for categorical variables. Appropriate statistical tests, such as T-tests or Chi-square tests, will be used to determine differences in baseline characteristics and sex differences where applicable. The normality of the variables will be explored using the Shapiro–Wilk test, and visual analysis with Q-Q plots and distribution of residuals will be applied. If the criteria associated with missing at random data are met, the relevance of the imputation will be evaluated.

For our primary outcomes, propensity analysis will be performed. This model allows us to reduce selection bias and strengthen inferences about the causal effect of an intervention in a non-randomized model (79). This analysis aims to balance the characteristics of the groups so that they are comparable in terms of all relevant variables, both known and unknown, that may influence the results. In addition, analysis of variance (ANOVA) or analysis of covariance (ANCOVA) will be applied depending on the objectives of the study. The inter-individual response of the participants will also be analyzed if deemed necessary. For our secondary objectives, we will analyze the possible mediating or moderating roles of our variables. Any additional analysis that the researchers consider necessary will be sought.

In parallel, sensitivity analyses will be performed according to the intention-to-treat principle. All analysis models will also adjust for relevant covariates such as sex, age, socioeconomic status, eating habits, smoking habits among others depending on their relevance in the model studied. To determine which variables are appropriate to use as covariates, directed acyclic graphs will be explored. These graphs are built on the expert knowledge of the researcher, which would facilitate the causal understanding of the phenomenon and the type of linkage between the variables involved, minimizing the introduction of bias during the design of the study and in the analysis of results (80, 81). All analyses will be performed in different software according to the needs of the researchers (e.g., R Studio, Jamovi, SPSS).

## Discussion

### Expected results and transfer to the work context

Reducing health problems associated with sedentary behavior is a crucial concern for governments worldwide (82). While several strategies are emerging to mitigate excessive sedentary time in desk-based workers, comprehensive research examining multiple outcomes and protocols is lacking (23, 24, 33). In this context, our study will compare two intervention types (B-B and C-P) that are equivalent in duration and intensity but differ in distribution. In this sense, our primary hypothesis postulates that both intervention groups obtain better results than the control group. However, we do not anticipate significant differences between the intervention groups due to their equal duration and intensity. This design, which is more realistic for future interventions, will allow us to compare (a) the effectiveness of a continuous break protocol versus an interval-based one and (b) the effectiveness of professional-directed pauses versus autonomous breaks directed by a computational application.

Furthermore, this research helps a gap in the literature by concentrating on three pivotal facets of participants’ health. Firstly, we hope that employing DXA for body composition measurement and accelerometry for assessing sedentary and physical activity time could enhance methodological precision in this field. Secondly, we aim to explore effective strategies for enhancing cardiometabolic risk markers in desk-based workers through a more comprehensive and innovative approach. Thirdly, our objective is to contribute to developing evidence-based interventions aimed at diminishing perceived stress levels and occupational pain experienced by workers. Addressing these three indicators could be significant for companies and public health policies. In this line, our hypothesis is that both intervention groups will yield better results compared to the control group.

However, this trial has limitations. First, randomization will not be possible due to the intervention’s characteristics as well as blinded allocation. Second, for the participants’ convenience, compositional measurements were scheduled in the afternoon. This may impact the measurements due to the participants’ hydration and food intake status. Nevertheless, in order to minimize the risk of measurement errors, particularly those related to the time of day, the same measurement protocol will be followed for all participants (83). Third, stress perceived by desk-based workers will be measured through a questionnaire, and exercise intensity will be gaged by RPE. In both instances, it would be desirable to incorporate complementary physiological indicators, such as cortisol levels and heart rate, respectively. Fourth, although the accelerometer allows to determine the sitting and break time, it is not completely appropriate to determine the stationary postures as other devices (e.g., inclinometers) (52). Additionally, our assessment of cardiometabolic risk factors does not encompass a lipid profile.

This study also has strengths to highlight. First, although the OSPAQ, DABQ, and single item of the physical activity questionnaire are not validated in Spanish language, our research group is actively working on these validations in a parallel study. Second, the intervention during 12 weeks already allows to observe behavioral change (44), which could extend in time the effects of the interventions carried out after the study’s end. Thirdly, integrating both device-based and traditional measures for cardiometabolic risk, physical activity, and sedentary lifestyle will enable the creation of a more comprehensive model to understand the effect of two protocols of active breaks at work in our outcomes. In this regard, it is important to remark on the use of DXA for body composition measurements, in contrast to the measures most commonly used in previous studies (i.e., BMI, waist circumference). Fourth, the determination of specific covariates that may affect our outcomes in important ways. These covariates will allow us to adjust our analysis model in order to comprehensively explore the effects of the intervention proposed in this study.

The results of this project can serve as a basis for future interventions, and government and company programs aimed at improving the health of desk-based workers. Promoting these strategies is essential, given that prolonged sitting in the workplace combined with physical inactivity is a crucial risk factor for workers’ physical, metabolic, and mental health.

In summary, “The UP Project” aims to identify effective ways to improve markers of cardiometabolic risk, perceived stress and occupational pain in office workers through breaks aimed at interrupting sedentary time during the workday. We hope that this effort will mark a breakthrough in the search for protocols to improve the health of office workers and contribute with scientific evidence that will contribute to the development of public policies to combat sedentary time.

## Ethics statement

The studies involving humans were approved by Ethics Committee of Pontificia Universidad Católica de Valparaíso (BIOEPUCV-HB 580–2023). The studies were conducted in accordance with the local legislation and institutional requirements. The participants provided their written informed consent to participate in this study. Written informed consent was obtained from the individual(s) for the publication of any identifiable images or data included in this article.

## Author contributions

CC-M: Conceptualization, Methodology, Project administration, Supervision, Writing – original draft, Writing – review & editing. RM-F: Conceptualization, Methodology, Project administration, Supervision, Writing – original draft, Writing – review & editing. JE-P: Methodology, Writing – review & editing. LF-R: Methodology, Writing – review & editing. NZ-C: Methodology, Writing – review & editing. IC-C: Methodology, Writing – review & editing. JL: Methodology, Writing – review & editing. GF: Methodology, Writing – review & editing. KS: Methodology, Writing – review & editing. JC-L: Methodology, Writing – review & editing. SH-J: Methodology, Writing – review & editing. TF: Methodology, Writing – review & editing. VL: Methodology, Writing – review & editing. FR-R: Methodology, Writing – review & editing. CB: Conceptualization, Methodology, Project administration, Supervision, Writing – original draft, Writing – review & editing.

## Footnotes

^1^ https://unchilemassano.minsal.gob.cl/
