# Supplementary material for: Study protocol and rationale of “the UP project”: evaluating the effectiveness of active breaks on health indicators in desk-based workers
Source: Front Public Health. 2024 Mar 19;12:1363015. doi: 10.3389/fpubh.2024.1363015 (PMC10985339; doi:10.3389/fpubh.2024.1363015)
Supplement: Supplementary file 1 [file Data_Sheet_1.DOCX]

**Satisfaction Questionnaire Active Face-to-Face Breaks**

Name:_____________________________________________________________________

Select the option based on your experience:

| *QUESTIONS* | **I don't agree at all (1)** | **I disagree (2)** | **Somewhat Disagree (3)** | **A little bit of agreement (4)** | **Okay (5)** | **I strongly agree (6)** |
| --- | --- | --- | --- | --- | --- | --- |
| Did you like the active pauses? | 1 | 2 | 3 | 4 | 5 | 6 |

|  | **YES (1)** | **NO (2)** |
| --- | --- | --- |
| Did you think about leaving the program at some point? | 1 | 2 |
| Would you keep breaks during your work routine during the year? | 1 | 2 |

|  | **Very Long (1)** | **Very Short (2)** | **Sufficient (3)** |
| --- | --- | --- | --- |
| Do you think the time allotted for the break was too long, too short or enough? | 1 | 2 | 3 |
|  | **Very mild (1)** | **Very High (2)** | **Sufficient (3)** |
| Do you think the intensity of the breaks was too slight, too high or enough? | 1 | 2 | 3 |
|  | **Many (1)** | **Few (2)** | **Enough (3)** |
| Do you think the number of days allocated to breaks during the week were many, few, or enough? | 1 | 2 | 3 |

|  | **I don't agree at all (1)** | **I disagree (2)** | **Somewhat Disagree (3)** | **A little bit of agreement (4)** | **Okay (5)** | **I strongly agree (6)** |
| --- | --- | --- | --- | --- | --- | --- |
| In general, did active breaks negatively disrupt your work? (e.g. you were in a meeting/task and had to interrupt it to your work) | 1 | 2 | 3 | 4 | 5 | 6 |
