# Supplementary material for: Study protocol and rationale of “the UP project”: evaluating the effectiveness of active breaks on health indicators in desk-based workers
Source: Front Public Health. 2024 Mar 19;12:1363015. doi: 10.3389/fpubh.2024.1363015 (PMC10985339; doi:10.3389/fpubh.2024.1363015)
Supplement: Supplementary file 2 [file Data_Sheet_2.DOCX]

**Satisfaction Questionnaire Active Breaks App**

Name:______________________________________________________

Select the option based on your experience:

| *QUESTIONS* | **I don't agree at all (1)** | **I disagree (2)** | **Somewhat Disagree (3)** | **A little bit of agreement (4)** | **Okay (5)** | **I strongly agree (6)** |
| --- | --- | --- | --- | --- | --- | --- |
| Did you like the pause app? | 1 | 2 | 3 | 4 | 5 | 6 |

|  | **YES (1)** | **NO (2)** |
| --- | --- | --- |
| Did you uninstall it or think about doing so? Yes/No | 1 | 2 |

|  | **Just stand up**  **(1)** | **Walking (2)** | **Do the exercises in the video (3)** | **Going Out for a Smoke**  **(4)** | **Other**  **(5)** |
| --- | --- | --- | --- | --- | --- |
| In general, what did you do during your 2 min break? | 1 | 2 | 3 | 4 | 5 |

|  | **Very Long (1)** | **Very Short (2)** | **Sufficient (3)** |
| --- | --- | --- | --- |
| Do you think the time allotted for the break was too long, too short or enough? | 1 | 2 | 3 |
